# Generation of Live Piglets for the First Time Using Sperm Retrieved from Immature Testicular Tissue Cryopreserved and Grafted into Nude Mice

**DOI:** 10.1371/journal.pone.0070989

**Published:** 2013-07-29

**Authors:** Hiroyuki Kaneko, Kazuhiro Kikuchi, Michiko Nakai, Tamas Somfai, Junko Noguchi, Fuminori Tanihara, Junya Ito, Naomi Kashiwazaki

**Affiliations:** 1 Animal Development and Differentiation Research Unit, National Institute of Agrobiological Sciences, Tsukuba, Ibaraki, Japan; 2 The United Graduate School of Veterinary Science, Yamaguchi University, Yamaguchi, Japan; 3 Transgenic Pig Research Unit, National Institute of Agrobiological Sciences, Tsukuba, Ibaraki, Japan; 4 Animal Breeding and Reproduction Division, NARO Institute of Livestock and Grassland Science, Tsukuba, Ibaraki, Japan; 5 Laboratory of Animal Reproduction, School of Veterinary Medicine, Azabu University, Sagamihara, Kanagawa, Japan; Institute of Zoology, Chinese Academy of Sciences, China

## Abstract

Cryopreservation of immature testicular tissues is essential for increasing the possibilities of offspring generation by testicular xenografting for agricultural or medical purposes. However, successful production of offspring from the sperm involved has never been reported previously. In the present study, therefore, using intracytoplasmic sperm injection (ICSI), we examined whether xenogeneic sperm obtained from immature pig testicular tissue after cryopreservation would have the capacity to produce live piglets. Testicular fragments from 9- to 11-day-old piglets were vitrified after 10- or 20-min immersion in vitrification solution containing ethylene glycol (EG), polyvinyl pyrrolidone (PVP) and trehalose as cryoprotectants, and then stored in liquid nitrogen for more than 140 days. Thirty nude mice were assigned to each immersion-time group. Testicular fragments were transplanted under the back skin of castrated mice immediately after warming and removal of the cryoprotectants. Blood and testicular grafts were then recovered from the recipient mice on days 60, 120, 180 and 230−350 (day 0 =  grafting). Histological assessment of the testicular grafts and analyses of inhibin and testosterone production revealed no significant differences between the two immersion-time groups, indicating equal growth activity of the cryopreserved tissues. A single sperm obtained from a mouse in each group on day 230−350 was injected into an *in vitro*-matured porcine oocyte, and then the ICSI oocytes were transferred to the oviducts of estrus-synchronized recipient gilts. One out of 4 gilts that had received oocytes fertilized using sperm from the 10-min immersion group delivered 2 live piglets, and one of another 4 gilts from the 20-min group delivered 4 live piglets. Thus, we have successfully generated porcine offspring utilizing sperm from immature testicular tissues after cryopreservation and transplantation into nude mice. The present model using pigs will be applicable to many large animals, since pigs are phylogenetically distant from the murine recipients.

## Introduction

Completion of spermatogenesis was first described by Honaramooz et al. [1] in testicular tissue of neonatal pigs and goats that had been grafted into nude mice. Since this pioneering study, fresh testicular tissues prepared from neonatal donors of many species have been grafted into immunodeficient mice, and in most cases the xenografts have initiated spermatogenesis, reaching the stage of elongated spermatids (primate [2], cattle [3]–[6], horse [7]) or sperm (primate [8], pig [1], , cat [14], [15], dog [16], rabbit [17]). Although limited so far to rabbit [17] or pig [13], intracytoplasmic sperm injection (ICSI) using xenogeneic sperm has been used successfully to producing live offspring. Thus, testicular xenografting makes it possible to obtain a new generation from young donors that have not yet reached sexual maturation and cannot be used for reproduction. In practice, however, testicular tissues may need to be stored until offspring production become necessary, and immediate testis transplantation is not always possible. Therefore, long-term preservation of testicular tissue that maintains its potential to produce sperm is desirable.

For this purpose, cryopreservation has been applied to neonatal or juvenile testicular tissues of several species such as human [18], [19], primates [20]–[22], pig [1], [9], [23], [24] and rabbit [17], and the ability of the cryopreserved tissue to complete spermatogenesis was assessed by xenografting into immunodeficient mice. Reports published so far have documented the capability of testicular tissues to grow in host mice and produce spermatocytes (human [19], primate [21]), spermatids (pig [9], [23]) or sperm (pig [1], [24], rabbit [17]) after they had been subjected to slow freezing using a low concentration of cryoprotectant such as dimethyl sulfoxide (DMSO), glycerol or ethylene glycol (EG). Vitrification, which employs high concentrations of cryoprotectants combined with an ultrahigh cooling rate, has also been shown to allow testicular tissue to produce spermatocytes (human [19]) or sperm (pig [24]). When applying these techniques for conservation of genetically valuable animals such as rare breeds, endangered species, or those with genetic modifications that result in neonatal lethality, there is a need to determine whether cryopreserved tissues grafted into immunodeficient mice produce sperm with full-term developmental ability. At present, there is no information about the developmental competence of these spermatogenic cells, although pups have been born using ICSI with sperm from cryopreserved tissues allografted into mice [17]. On the other hand, our group has explored cryopreservation of porcine oocytes and zygotes by vitrification. Oocytes vitrified at the germinal vesicle stage maintained developmental competence to the blastocyst stage after maturation and fertilization *in vitro* [25]. Live piglets were successfully produced from *in vitro*-produced zygotes vitrified at the pronuclear stage [26]. These previous findings indicate that our vitrification method allows female germ cells to maintain full developmental competence, thus raising the possibility that it might be applicable to cryopreservation of testicular tissue.

In the present study, therefore, we used pigs as a large mammal model to examine whether our vitrification protocol could be modified to preserve immature testicular tissue with complete spermatogenic potential. Development of the grafted tissue was evaluated histologically and endocrinologically. Lastly, we injected sperm recovered from recipient mice into *in vitro*-matured porcine oocytes and assessed the full-term fetal development of the fertilized oocytes after transfer to estrus-synchronized recipients.

## Materials and Methods

All chemicals were purchased from the Sigma-Aldrich Corporation (St. Louis, MO, USA), unless otherwise indicated.

### Ethics statement

Protocols for the use of animals were approved by the Animal Care Committee of the National Institute of Agrobiological Sciences (ID: H18-008-2). All surgery was performed under anesthesia with sodium pentobarbital and ether, and all efforts were made to minimize suffering.

### Animals

Nine crossbred (Landrace×Large White×Duroc) piglets aged 9 to 11 days, born at the National Institute of Livestock and Grassland Science, were used as donors. Sixty male immunodeficient mice (Crlj:CD1-*Foxn*1*^nu^*; Charles River Japan, Yokohama, Japan) aged at 5−6 weeks were used as recipients for the xenografts. They were kept in an environmentally controlled room (Koito, Yokohama Japan) maintained at a temperature at 24°C and humidity at 50% and illuminated daily from 05:00 to 19:00 h. Food was provided at *ad libitum*. Eight gilts (Landrace×Large White crossbreed), purchased commercially (CIMCO, Tokyo, Japan), were used as recipients for transfer of fertilized oocytes after ICSI. The gilts were housed individually and fed *ad libitum*.

### Experimental design

High concentrations of cryoprotectants in vitrification solution, combined with an ultrahigh cooling rate, prevent the formation of intracellar ice but have toxic effects on cells [27]. Therefore neonatal porcine testes were vitrified after immersion for different periods (10 or 20 min) in vitrification solution to estimate the most appropriate duration of exposure to cryoprotectants. Recipient mice after castration were divided into two groups: 30 mice received testicular fragments that had been vitrified after 10 min of immersion (10-min immersion group) and another 30 mice received fragments after 20 min of immersion (20-min immersion group). The mice in each group were killed with an ether overdose for sampling at 60, 120, 180 or 230−350 days after grafting (day 0 =  grafting). Testis development was then compared between the two groups. The other 7 mice were used for collection of blood samples under castrated and ungrafted situations. For assessment of the developmental ability of porcine sperm recovered from mice, four gilts were assigned to each group for receiving fertilized oocytes produced by ICSI using the xenogeneic sperm.

### Vitrification, storage and warming of testicular tissue

Immediately after removal of the testis, the cortex was cut into small pieces, which were further minced into pieces measuring approximately 1.5×1.5×1.5 mm in saline supplemented with 668 units/ml penicillin G potassium and 0.2 mg/ml streptomycin sulfate. Testicular fragments were vitrified according to the method described by Dinnyes et al. [28] and Somfai et al. [29], with some modifications. Briefly, approximately 30 fragments were washed two times in 2 ml of base solution (BS; modified North Carolina State University (NCSU)-37 solution without glucose but supplemented with 0.17 mM sodium pyruvate, 2.73 mM sodium lactate, 4 mg/ml BSA, and 50 µM β-mercaptoethanol, 100 units/ml penicillin G potassium, and 0.1 mg/ml streptomycin sulfate (antibiotics) (IVC-PyrLac; [30]) supplemented further with 20 mM HEPES (Dojindo, Kumamoto, Japan), and then transferred into 500 μl of equilibration solution (BS supplemented with 4% ethylene glycol (EG) in four-well dishes (Nunclon Multidishes; Thermo Fisher Scientific, Waltham, NA, USA) and incubated for 15 min at room temperature. Equilibrated fragments were immersed in 500 μl of vitrification solution (BS supplemented with 35% EG, 5% polyvinyl pyrrolidone (PVP; Mr 40000) and 0.3 M trehalose) for 10 or 20 min. Each fragment was aspirated into a glass capillary, and dropped onto an aluminum foil boat partially immersed in liquid nitrogen (LN_2_) with minimum volume of vitrification solution to form a micro-droplet. Finally, the vitrified droplets were placed in 2-ml cryotubes (Iwaki, Tokyo, Japan) immersed in LN_2_ using cooled forceps, and then the cryotubes were plunged into LN_2_. After storage for at least 140 days, Vitrified droplets containing testicular fragments were unloaded from the cryotubes onto an aluminum boat partially immersed in LN_2_. They were then transferred to a warming solution (BS supplemented with 0.4 M trehalose) set at 37°C and incubated in it at 37°C for 2 min. The fragments were consecutively transferred for 2-min periods into 500 μl of BS supplemented with 0.2 M, 0.1 M, and 0.05M trehalose at room temperature, then finally incubated in saline supplemented with antibiotics for 2 min at room temperature.

Six fragments before washing with base solution and just after cryopreservation-warming, prepared from 4 respective donors, were fixed in Bouin’s solution for histological examination.

### Testicular tissue xenografting

Xenografting of testicular fragments after cryopreservation and warming was carried out based on the methods described previously [11]. Mice were anesthetized with a combination of pentobarbital sodium (Somnopentyl; Kyoritsu, Tokyo, Japan) and ether, and then castrated. Immediately after castration, 30 testicular fragments were inserted under the skin of the back within 10 min after the end of warming.

### Collection of testicular tissue and blood from recipient mice

All visible testicular grafts were recovered in collection medium (Dulbecco’s PBS; Nissui, Tokyo, Japan, supplemented with 5 mg/ml BSA) at 37°C and weighed. Three pieces were excised from the different larger grafts in each mouse and fixed in Bouin’s solution for histological examination. The remaining portions were used to recover sperm. Serum samples from the recipient mice were stored at −30°C until immunoassay to estimate the production of inhibin and testosterone in the grafted tissues.

### Collection of sperm from xenografts

The recovered grafts were minced in collection medium, and the presence of sperm released into the medium was recorded. For ICSI, testicular grafts that had been recovered from mice on day 230−350 were used, since the percentage of mice that produced porcine sperm increased with time after grafting (see Results). The tissue suspension was centrifuged for 10 min at 600×*g*, and the supernatant was discarded. Washing was repeated 3 times. The pellet was resuspended in collection medium and maintained at room temperature until use for ICSI.

### Oocyte collection and *in vitro* maturation

Ovaries were obtained from prepubertal crossbred gilts (Landrace×Large White×Duroc breeds) at a local slaughterhouse, and transported to the laboratory at 35°C. Cumulus-oocyte complexes (COCs) were collected from follicles 2−6 mm in diameter in TCM 199 (with Hanks’ salts) supplemented with 10% (v/v) fetal bovine serum (Gibco, Carlsbad, CA, USA), and antibiotics. *In vitro* maturation was performed as reported previously [30]. Briefly, about 40 COCs were cultured in 500 μl of maturation medium for 20−22 h in four-well dishes. The medium employed was NCSU-37 solution containing 10% (v/v) porcine follicular fluid, 0.6 mM cysteine, 50 µM β-mercaptoethanol, 1 mM dibutyl cAMP (dbcAMP), antibiotics, 10 IU/ml eCG (Serotropin; ASKA, Tokyo, Japan), and 10 IU/ml hCG (Puberogen; Novartis Animal Health, Tokyo, Japan). The COCs were subsequently cultured for 24 h in maturation medium without dbcAMP and hormones. Maturation culture was carried out at 39°C under conditions in which CO_2_, O_2_, and N_2_ were adjusted to 5%, 5%, and 90%, respectively (5% O_2_ conditions). After culture, cumulus cells were removed from the oocytes by treatment with 150 IU/ml hyaluronidase and gentle pipetting. Denuded oocytes with the first polar body were harvested under a stereomicroscope, and used as *in vitro*-matured oocytes.

### ICSI and oocyte stimulation

ICSI was carried out as described previously [12], [13], . Two solutions were prepared for ICSI: 1) for oocytes, IVC-PyrLac and IVC-PyrLac supplemented with 20 mM Hepes, the osmolality being adjusted to 285 mOsm/kg (IVC-PyrLac-Hepes; [12], [13], ), and 2) for sperm, IVC-PyrLac-Hepes supplemented with 4% (w/v) PVP (Mr 360000) (IVC-PyrLac-Hepes-PVP). Sperm were injected as described previously [12], [13]. Approximately 20 *in vitro* oocytes were transferred to a 20-μl drop of IVC-PyrLac-Hepes. The solution containing the mature oocytes was placed on the cover of a plastic dish (Falcon 35–1005; Becton Dickinson and Company, Franklin Lakes, NJ, USA). A small volume (0.5 μl) of the sperm suspension was transferred to a 2-μl drop of IVC-PyrLac-Hepes-PVP, which was prepared close to the drops used for the oocytes. All drops were covered with paraffin oil (Paraffin Liquid; Nacalai Tesque, Kyoto, Japan). A single sperm was aspirated tail first from the suspension into the injection pipette, and the pipette was moved to the drop containing the oocyte. The sperm was injected into the ooplasm using a Piezo-actuated micromanipulator (PMAS-CT150; Prime Tech, Tsuchiura, Japan). One hour after the injection, the sperm-injected oocytes (20 in all) were transferred to an activation solution consisting of 0.28 M D-mannitol, 0.05 mM CaCl_2_, 0.1 mM MgSO_4_, and 0.1 mg/ml BSA and washed once. They were then stimulated with a direct current pulse of 1.5 kV/cm for 20 μsec using a somatic hybridizer (SSH-10; Shimadzu, Kyoto, Japan). Parthenogenetic oocytes for assisted pregnancy were generated by electrostimulation with a direct pulse of 2.2 kV/cm for 30 μsec and then incubated in 10 μg/ml cytochalasin B for 3 h.

### Transfer of xenogeneic sperm-injected oocytes

Estrus synchronization of the recipient gilts was induced by an i.m. injection of 1000 IU of eCG, followed 72 h later by an injection of 500 IU of hCG. Ovulation was expected at 40−45 h after the hCG injection. Mixtures of 60−100 fertilized oocytes and approximately 30 parthenogenetic oocytes were surgically transferred to both oviducts of recipient gilts. Pregnancy was diagnosed in the recipients using an echographic pregnancy detector (Fujihira, Tokyo, Japan).

### Histological analysis of testicular tissue

To compare the status of spermatogenesis, we collected three pieces of tissue from different portions of the left testis of 4 crossbred (Landrace×Large White) boars between 9 and 12 months of age at a local abattoir. Testicular fragments before transplantation and those excised from the grafts in mice and from young mature boars were embedded in paraffin, and then were cut into sections 6 μm thick. The sections were then stained with hematoxylin and eosin. The seminiferous tubule cross-sections were sorted into the following categories: 1) no germ cells present (tubule cross-sections showing Sertoli cells only); 2) gonocytes/spermatogonia present; 3) spermatocytes present as the most advanced germ cells; 4) round spermatids present as the most advanced germ cells; 5) elongated spermatids present as the most advanced germ cells; and 6) mature sperm present in the lumina of the tubules in which elongated spermatids coexisted. All seminiferous tubules observed in each cross-section of testicular fragments from each animal were examined. The data obtained from all fragments from each animal were pooled, and the percentage of tubule cross-sections in each category relative to the total number of tubule cross-sections examined was calculated.

### Fluoroimmunoassays (FIAs) for inhibin and testosterone

Concentrations of inhibin (total inhibin) in serum were determined by a competitive immunoassay using europium (Eu)-labeled bovine inhibin A as a probe [34]. In time-resolved FIA (Tr-FIA) of bovine total inhibin, antibovine inhibin serum (TNDH-1; [35]), provided by Dr. K. Taya of Tokyo University of Agriculture and Technology, Fuchu, Tokyo, Japan, was used as a primary antibody. Bovine 32-kDa inhibin A, purified from follicular fluid, was provided by Dr. Y. Hasegawa of Kitasato University, Towada, Aomori, Japan. The detection limit of the FIA was 0.078 ng/ml. The intra- and interassay CVs were 9.3% and 16.8%, respectively.

The concentrations of testosterone were determined with a commercial competitive FIA kit (DELFIA testosterone kits, PerkinElmer Japan, Yokohama, Japan). Testosterone was extracted from the serum with 2 ml of ether before being applied to the kit. The detection limit of the FIA was 1.0 pg/ml. The intraassay and interassay CVs were 10.9% and 15.1%, respectively.

### Data analyses

The mean weight of five sets of 30 fragments before grafting was 34.1±1.0 (± SEM) mg (approximately 1 mg per fragment). When the total weight of grafted tissues recovered from a single mouse exceeded the 95% confidence limit (32.1−36.1 mg), porcine testicular tissues were judged to have grown in the mouse, and data obtained from such mice were subjected to statistical analyses. Data on the development and hormone production of testes in each group were subjected to one-way ANOVA. The effects of immersion time on testis development and hormone production were analyzed by two-way ANOVA. When a significant effect was detected by ANOVA, the difference between two means was determined by Student’s *t* test and differences among more than two means were determined by Tukey’s test. The General Linear Models of Statistical Analysis Systems, ver 9.2 (SAS Inc., Cary, NC, USA) was used for these analyses. Differences at P<0.05 were considered significant.

## Results

### Development of cryopreserved testicular tissues

**Figure 1:**
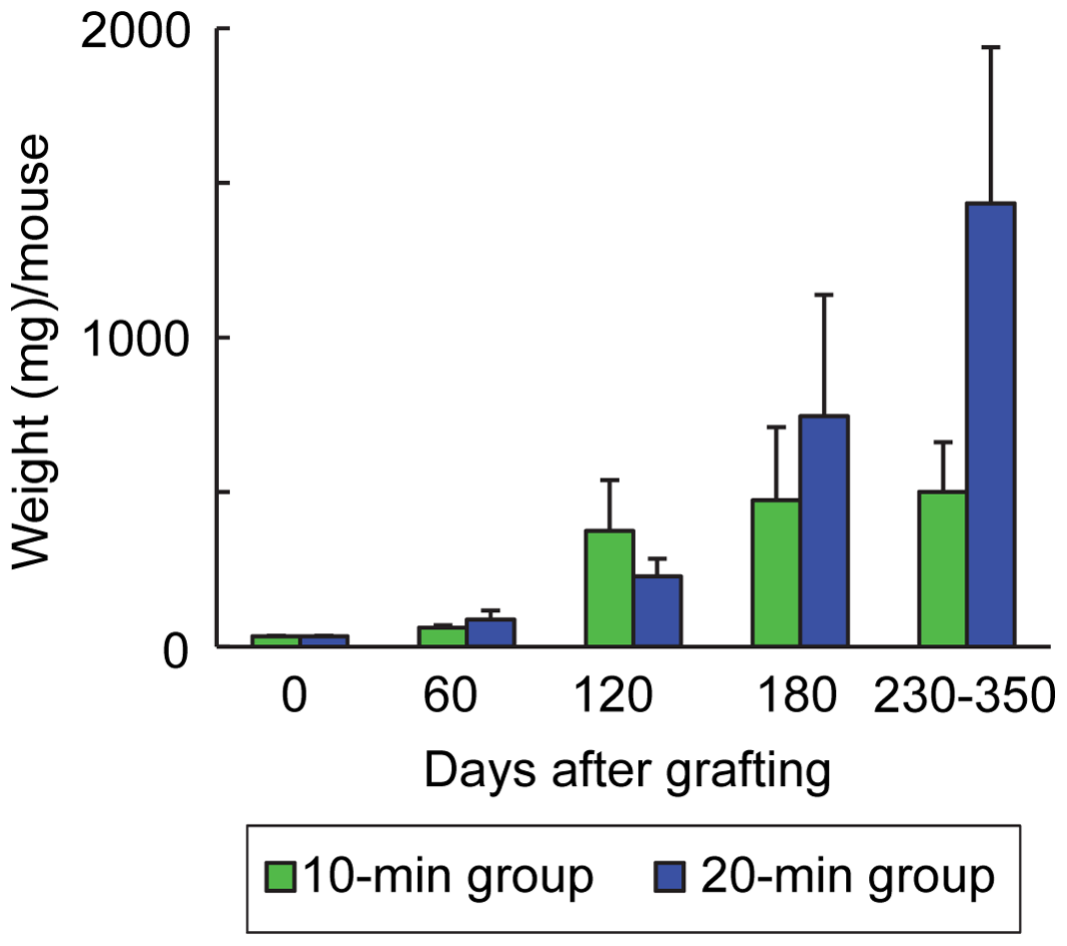
Weights of cryopreserved porcine testicular tissue in mice. All visible grafts were recovered from the recipient mice, and values are means of total weights per mouse (± SEM) for 4 to 7 mice in each group.

**Figure 2:**
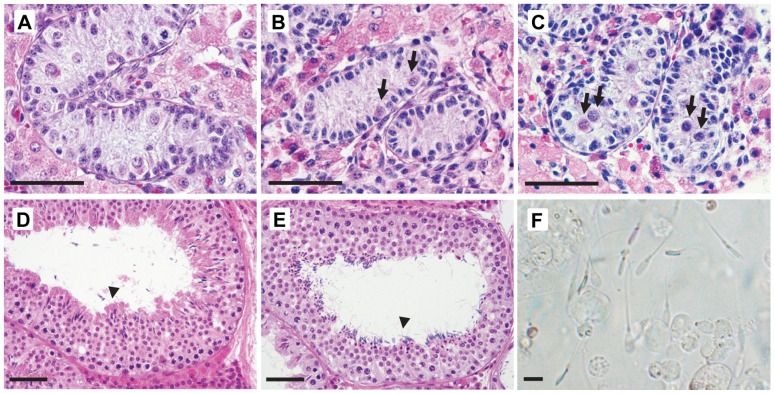
Histological appearance of cryopreserved porcine testicular tissues before and after xenografting, and of recovered sperm. (A) Fresh testicular tissue from a 9-day-old piglet before rinsing with base solution. Tissues were vitrified after being immersed in vitrification solution for 10 or 20 min (10- or 20-min group), stored in LN_2_ and then warmed: tissue in the (B) 10- and (C) 20-min group contained distinct gonocytes (arrows). Tissue recovered from mice in the (D) 10-min and (E) 20-min groups on day 230−350 contained elongated spermatids and sperm (arrow heads). Bars  = 50 μm. (E) Sperm retrieved from xenografts on days 230−350. Bar  = 10 μm.

**Figure 3:**
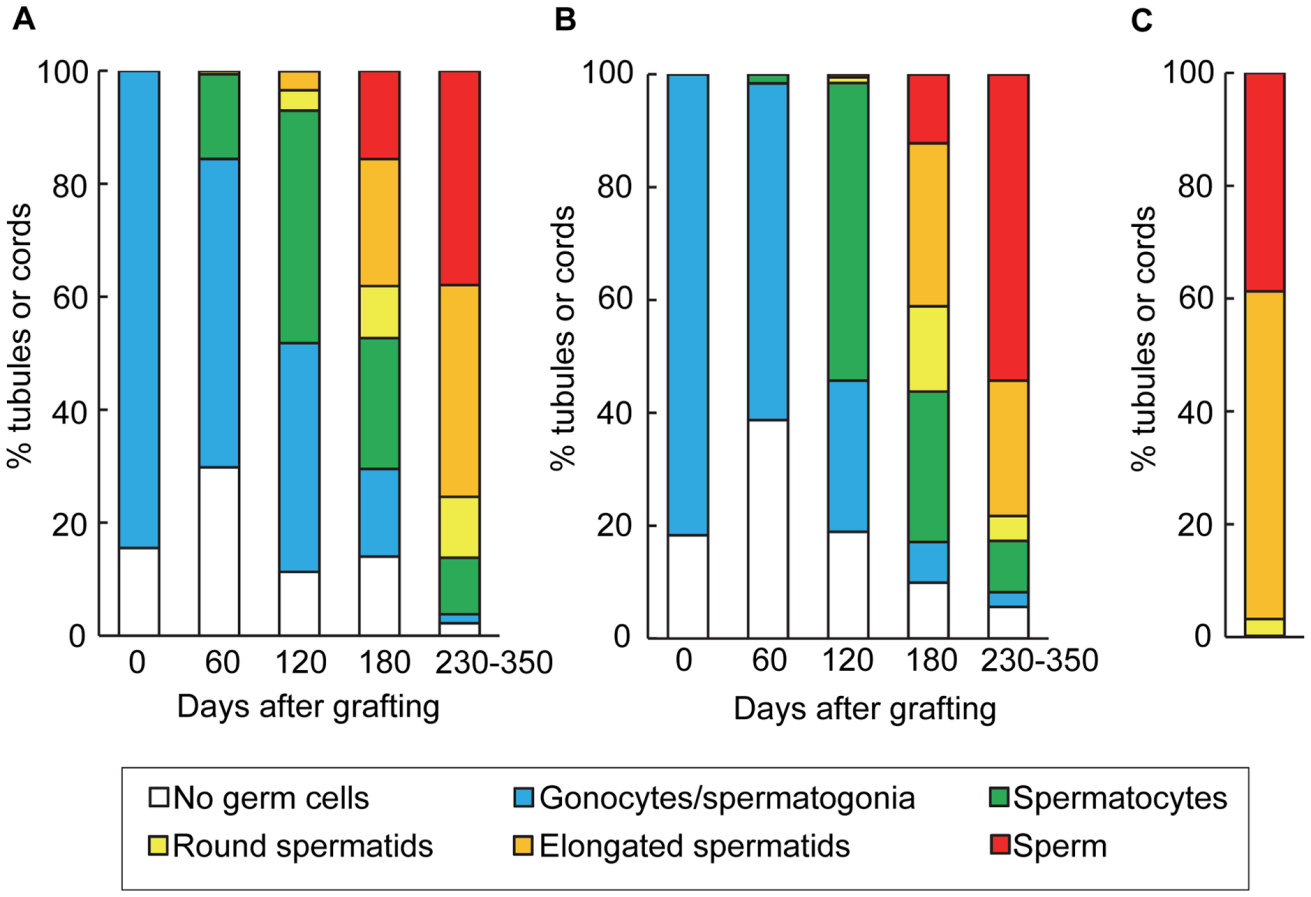
Differentiation of seminiferous tubules in cryopreserved porcine testicular tissues grafted into mice. Percentages of seminiferous cord or tubule cross-sections in (A) the 10-min or (B) 20-min group, as classified by the most advanced type of germ cell present, are shown. (C) Percentages of seminiferous tubule cross-sections in 9- to 12-month-old boars, classified according to the same criteria, are also presented. Data were obtained from four animals.

Growth of porcine testicular tissue, judged using the criteria explained in the data analysis section, was recorded in 20 of 30 mice in each group; the other 20 mice in both groups showed no testicular growth or died before sampling. Weights of all visible grafted tissues per mouse had increased (P<0.05) up to 500 mg by day 230–350 in each immersion-time group (Fig. 1) from 34.1±1.0 (mean ± SEM) mg on day 0 (day 0 =  grafting). There were no significant differences in testicular weights between the two groups (P = 0.08). Compared to the morphology of tissue before treatment with base solution (Fig. 2A), no clear structural degradation, with distinct gonocytes, was observed in the tubules after cryopreservation and warming (Figs. 2B and C). Prior to grafting, testicular tissues contained seminiferous cords consisting of only gonocytes/spermatogonia or Sertoli cells (Figs. 2B and C and Figs 3A and B). In both groups, spermatocytes first appeared on day 60 and then spermatids on day 120 (Figs. 3A and B). Significant (P<0.05) increases in the percentage of tubule cross-sections containing elongated spermatids or sperm were noted on day 180 (elongated spermatids; 22.5±8.7% and 28.9 ±8.1% in the 10- and 20-min groups, respectively, sperm; 15.6±6.1% and 12.2±5.8%, respectively) compared with each value on day 120 (elongated spermatids; less than 3% in each group, sperm; less than 0.1%). On day 230−350, the ratio of tubules with sperm had further increased (P<0.05) to 35% (Figs. 2D and E and Figs. 3A and B). There were no significant differences between the two immersion-time groups in the percentage of tubules in each category (P>0.08). When compared with the differentiation statuses of seminiferous tubules in young adult boars, the proportion of tubules showing spermatogenesis to stages of elongated spermatids and sperm was lower (P<0.05) in the grafts on day 230−350 (approximately 75% in the grafts in the two groups versus 96% in boars), while higher (P<0.05) percentages of tubules contained no germ cells or spermatogonia in the grafts (approximately 7% in the grafts versus 0.1% in boars) (Fig. 3). On day 120, sperm were recovered from the grafts from 2 out of 5 mice in the 10-min group and 1 of 5 mice in the 20-min group. The recovery rate increased with time after grafting, being 4/5 and 3/5 mice on day 180 and 8/8 and 11/17 mice on day 230−350 in the 10- and 20-min groups, respectively (Fig. 2F).

### Hormonal profiles in mice

**Figure 4:**
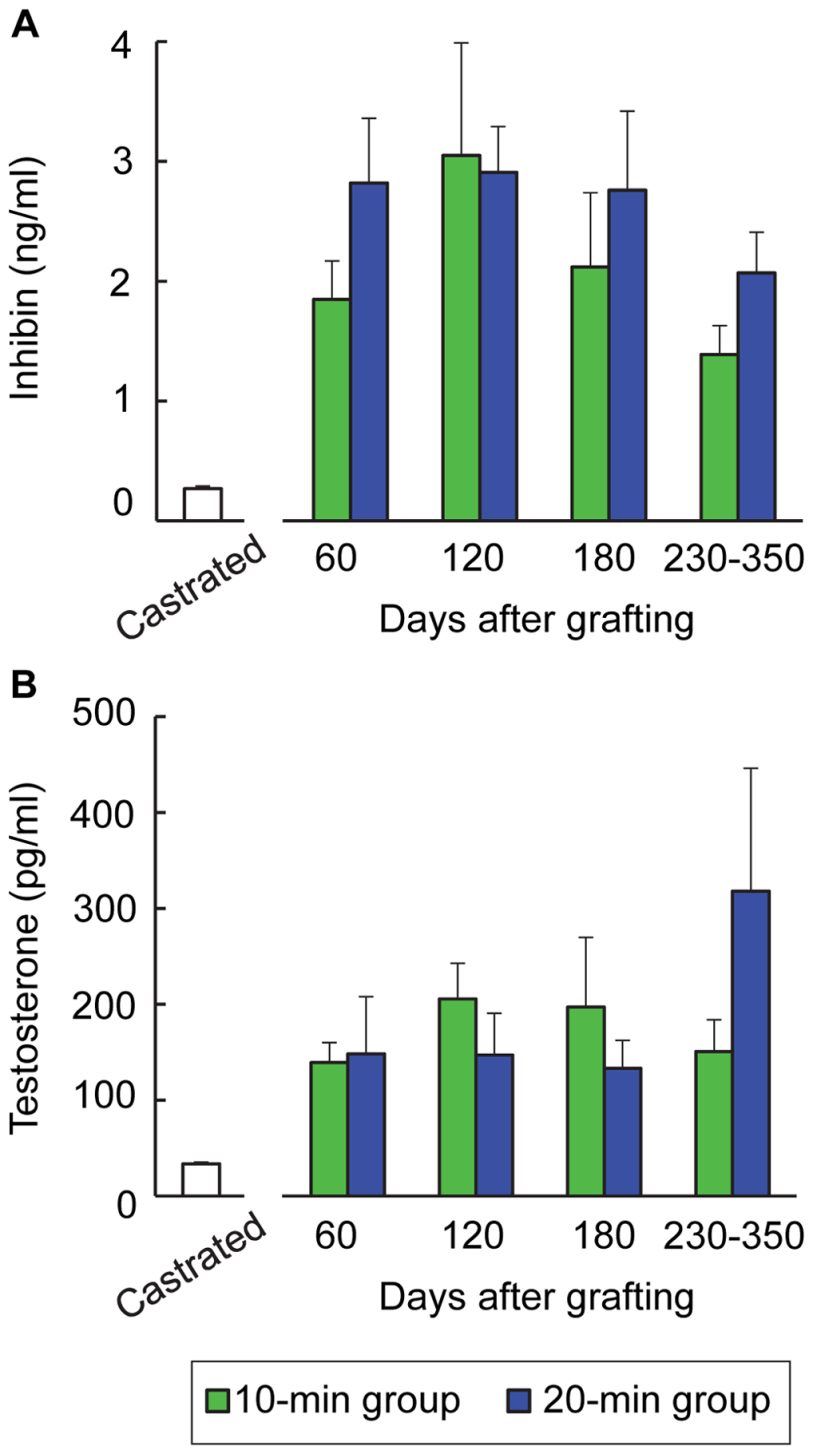
Hormonal profiles of the recipient mice. Serum concentrations of (A) inhibin and (B) testosterone in the recipient mice after castration and after grafting of cryopreserved porcine tissue of the 10- and 20-min groups are shown. Values are means ± SEM for 4 to 7 mice.

The serum concentrations of inhibin and testosterone were 0.27±0.02 ng/ml and 33.7±1.7 pg/ml in the castrated and ungrafted mice, respectively (Figs. 4A and B). After grafting of porcine testicular tissues, the concentrations of both inhibin and testosterone increased significantly (P<0.05) in each group, but no differences were noted in the concentrations of the two hormones between the two groups.

### Developmental ability of porcine sperm retrieved from mice

**Figure 5:**
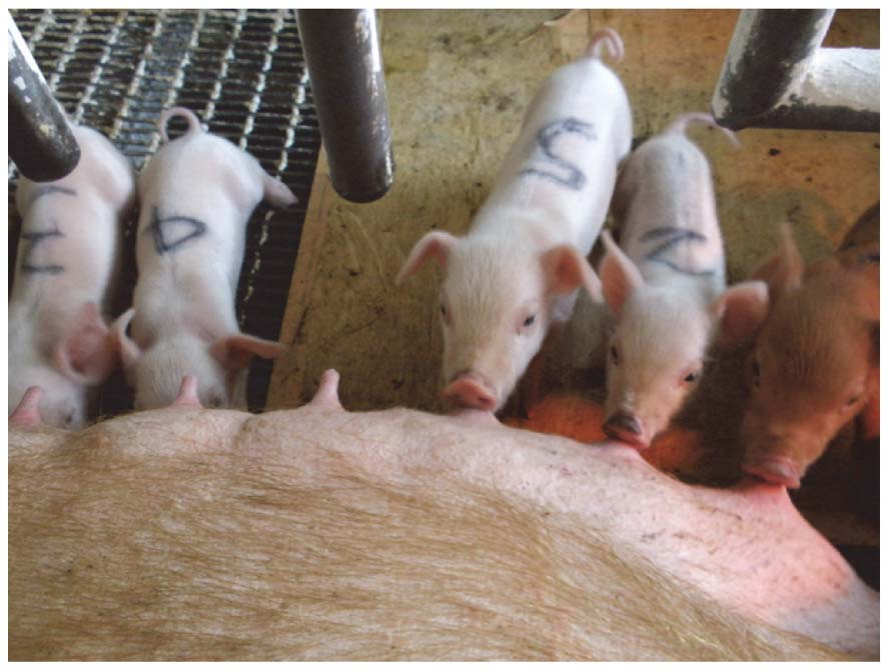
Generation of offspring after ICSI using sperm from cryopreserved porcine testicular xenografts. Two male and 3 female piglets were born after transfer of fertilized oocytes produced by ICSI using sperm recovered from a mouse on day 254 in the 20-min group. Tissue was stored in LN_2_ for 188 days. Photographed on the fifth day after birth.

Two out of eight recipient gilts gave birth (Table S1): one delivered 1 male piglet (body weight: 1.6 kg) and 1 female piglet (1.2 kg) at 108 days after transfer of the oocytes injected with sperm from a mouse in the 10-min immersion group, and the other 2 male piglets (0.7 and 0.7 kg) and 3 female piglets (0.7, 0.7 and 0.9 kg) at 112 days in the 20-min group (Fig. 5). These piglets subsequently developed normally, their body weights reaching 7.3±0.6 kg at the age of 30 days.

## Discussion

Xenografting of immature testicular tissue combined with cryopreservation is expected to allow rescue and conservation of genetic resources for rare animals, since this combination allows sperm to be obtained when offspring production is desirable, from genetically valuable males if they die before reaching sexual maturation. To realize the potential application of these techniques, it should be demonstrated that sperm derived from cryopreserved testicular tissues have the ability to develop into viable offspring. To our knowledge, this is the first reported study to have successfully produced live offspring by ICSI using sperm from testicular xenografts after long-term cryopreservation.

It has been proven that vitrification is a potent method for cryopreserving testicular tissue so that it maintains its ability to initiate [19] or complete spermatogenesis [24]. In vitrification, combined usage of cryoprotectants with low molecular weights and high penetration rates, such as DMSO, EG and glycerol, and those with higher molecular weights protecting the cell membrane, such as sugars and PVP, is popular. Previous studies have applied a mixture of DMSO, EG and sucrose, or of glycerol, EG and sucrose, to vitrification of testicular fragments from humans [19], primates [22] and pigs [24]. However, in the present study, we chose another vitrification solution containing EG, PVP and trehalose, which had been used successfully for porcine oocytes and zygotes [25], [26], and examined the most appropriate period of exposure to vitrification solution, in order to reduce the toxic effects of cryoprotectants. Taking into account the size of the pig testicular fragments, our immersion time for 1 mg of tissue (10 or 20 min) was relatively longer than that used for 5−30 mg tissue (5 or 15 min) in the previous study [24]; this indicates that the optimal exposure period depends on the combination of cryoprotectants present in the vitrification solution.

The present histologic examinations revealed no differences in the differentiation of seminiferous cord/tubules between the two immersion-time groups, being consistent with changes in the weights of grafted tissue. No difference between the two groups was also noted in the serum profiles of inhibin and testosterone in the recipient mice. Sertoli and Leydig cells are the major sources of testosterone [36], [37] and inhibin [38], [39] production in pigs, respectively. We conclude that the growth activities of grafted tissues were equivalent after vitrification using immersion times of 10 and 20 min. Chronologically, the proportions of seminiferous tubules containing elongated spermatids and/or sperm showed apparent increases from day 180, concomitant with an increased proportion of sperm recovered from the recipient mice. This indicates that the ability of grafted tissue to produce sperm increased with time after xenografting: similar time-dependent changes were reported in the previous study xenografted fresh testicular tissue [11], [14], [24]. Although the numbers of sperm recovered from xenografts on days 230−350 were sufficient for subsequent ICSI to produce approximately 100 fertilized oocytes for transfer to recipients, spermatogenesis in the grafted tissue did not reach the quantitatively normal levels observed in young adult boars of the same age. Grafted tissues had a lower proportion of seminiferous tubule cross-sections containing elongated spermatids or sperm, and a higher percentage of tubules with no germ cells or spermatocytes, compared with intact testes. Damage resulting from vitrification and cryopreservation, and ischemia after subcutaneous grafting, are likely to affect the development of grafted tissues. Interestingly, cryopreservation has been reported to induce the accumulation of hydrogen peroxide in mouse embryos [40] and porcine oocytes [29]. Oxidative stress may be a factor influencing the developmental ability of testicular tissues.

Like our previous study in which fertilized oocytes were produced by ICSI using sperm from fresh xenografts [13], 50−100 fertilized oocytes and 30−40 parthenogenetic oocytes were simultaneously transferred into the oviducts of the recipient gilts for the following reasons. It is generally accepted that pigs require a minimum of about 4 embryos in the uterus for maintenance of pregnancy [41]. Our previous study [31] suggested that porcine fertilized oocytes produced by ICSI have lower viability than conventionally *in vitro*-fertilized oocytes [30]. Co-transfer of parthenogenotes with a single embryo was proven to be sufficient for maintenance of pregnancy and development of the embryo [42]. Through co-transfer, we were able to obtain live piglets from 4 recipient gilts in each immersion-time group; these results clearly indicate that our vitrification protocols with 10 or 20 min of exposure to cryoprotectant are effective for cryopreservation of testicular tissues, and allow the full developmental competence of germ cells to be maintained. In the present study, live offspring were obtained using sperm from testicular tissues after preservation for 6 months, but not sperm from testis after preservation for 1.5 years (Table S1). Because of the relatively small number of recipients used, it is difficult to draw a firm conclusion; therefore, further long-term preservation will be necessary for field application.

To date, pups have been obtained using sperm from cryopreserved testicular tissue allografted into mice [17]. Use of a pig model, rather than mice, enhances the importance of this study, since pigs are phylogenetically distant from the murine recipients. Application of testicular xenografting has proven effective for salvaging genetic information from immature males of many valuable species and for recovery of the donor genetic pool, if appropriate artificial reproductive technologies, such as *in vitro* maturation/fertilization, and ICSI, are prepared. Testicular xenografting combined with cryopreservation will be useful not only for conservation of genetic resources but also for progress in medical research. In general, there is a possibility that genetically manipulated animals might have certain reproductive disadvantages, such as a degree of male infertility, or the presence of neonatal lethal traits [43], [44]. Recently, porcine models of severe combined immunodeficiency [45] and hemophilia A [46] were generated by cloning technology. However, disruption of X-linked genes encoding the interleukin-2 receptor (SCID model) or coagulation factor VIII (hemophilia model) caused neonatal death in cloned male pigs owing to infections and hemophilia. Xenografting of cryopreserved testicular tissue may contribute to the conservation and distribution of these model animals. An additional merit of this study is that vitrification methods are easy to perform and do not require a program freezer.

In conclusion, our present findings have demonstrated another vitrification protocol that allows porcine testicular tissue to maintain its ability to produce sperm after xenografting. Sperm retrieved from porcine xenografts were proven to have full developmental ability, developing into live piglets after ICSI.

## Supporting Information

Table S1 **Transfer to synchronized recipients of porcine oocytes injected with sperm from cryopreserved xenografts.**
(DOC)
