# Supplementary material for: Generation of Live Piglets for the First Time Using Sperm Retrieved from Immature Testicular Tissue Cryopreserved and Grafted into Nude Mice
Source: PLoS One. 2013 Jul 29;8(7):e70989. doi: 10.1371/journal.pone.0070989 (PMC3726602; doi:10.1371/journal.pone.0070989)
Supplement: Table S1 — Transfer to synchronized recipients of porcine oocytes injected with sperm from cryopreserved xenografts. (DOC) [file pone.0070989.s001.doc]

Table S1. Transfer to synchronized recipients of porcine oocytes injected with sperm from cryopreserved xenografts.

| Immersion-time group | Recipient No. | Preservation of testicular tissue before grafting (days) | Sperm collection (days postgrafting) | No. of fertilized oocytes transferred | Pregnancy | No. of piglets born |
| --- | --- | --- | --- | --- | --- | --- |
| 10-min | 1  2  3  4 | 140  188  585  587 | 231  230  318  234 | 74  70  100  101 | +  ‒  ‒  ‒ | ♂;1, ♀;1 |
| 20-min | 1  2  3  4 | 188  188  578  587 | 230  254  291  248 | 59  89  79  97 | ‒  +  ‒  ‒ | ♂;2 ♀;3 |
